# Supplementary material for: Acceptance and compliance with micronutrient powder (MNP) among children aged 6–23 months in northern Nigeria
Source: PLOS Glob Public Health. 2022 Oct 17;2(10):e0000961. doi: 10.1371/journal.pgph.0000961 (PMC10022258; doi:10.1371/journal.pgph.0000961)
Supplement: S1 Dataset — (ZIP) [file pgph.0000961.s008.zip › S8_Dataset/Direct Observations.zip!/Direct Observations/070715_A_GA_AD_2_DO.pdf]

1.1.1 Instrument F –Direct Observation Form

<<Instrument F Direct Observation Form>>
**PI Name:** PragyaMathema,Stephen Kodish<br>**Study Title:** Formative research to develop an infant and young child feeding (IYCF) intervention with micronutrient powder (MNP) for children aged 6 – 23 months<br>**PI Version/Date:** Version1.0/January 24, 2015

Date: ___7/07/2015_____Location: _ganwoki community in Ganye____

Data Collector: Aish aliyu dahiru__________Participant ID: A mud house with a well in front___

Main goals of this direct observation are to answer the following questions:<br><br>1. In <u>this</u> household, what would be the best way(s) to give nutritional supplements to <u>young children</u>? Why?<br><br>2. In <u>this</u> household, describe the food sharing that you observe throughout the meal. Are there any <u>predictable</u> patterns?<br><br>3. In <u>this</u> household, who is favored and how is this favoritism expressed during meal preparation and eating?

(TEAR OFF AT PERFORATED DOTTED LINE BELOW)

PARTICIPANT ID: _________

Household Composition Table (Fill out <u>as</u> you do data collection)

INITIALS	SEX (M/F)	AGE (estimated)	Relationship to household	Description
M	F	27Yrs	mother	Wearing pink and blue wrapper
C	M	13month(observe child)	Baby (son)	
OG	F	14 yrs	Older daughter	Wearing blue wrapper and red shirt
HB	M	37yrs	Husband brother	Wearing brown kaftan
G	F	17yrs	Mother sister	Wearing brown shirt and skit
OB	M	5yrs	Older son	Wearing blue checked shirt and black short uniform
H	M	34yrs	husband	Wearing brown trouser and yellow shirt

PARTICIPANT ID: __________

TIME	ACTOR INITIALS	RECIPIENT INITIALS	WHAT HAPPENED	FOOD	Reactivity
8:00	M		sweeping		
8:05	M		sweeping		
8:05	C		Playing in the verandah		
8:10	M		sweeping		
8:15	M		Washing hand to carry baby		
8:20	M	C	Carry child on her laps		
8:25	M	C	Breastfeeding the child		
8:30	OG		Washing the utensils		
8:35	M	C	Breastfeeding the child		
8:40	OG		Washing the utensils		
8:45	M	C	Breastfeeding the child		
8:50	OG		Taking the cleaned utensils into the room		
8:55	M		Starting fire		
9:00	M	OG	Starting fire		
9:05	M	OG	Chatting with older daughter sitting under the shade beside the room		
9:10	M	OG	Chatting with older daughter waiting for water to boil		
9:15	M	C	Carrying the child on her laps		

TIME	ACTOR INITIALS	RECIPIENT INITIALS	WHAT HAPPENED	FOOD	Reactivity
9:20	M	C	Breast feeding the child		
9:25	M	C	Breast feeding the child		
9:30	C		Sleeping on the mothers lap		
9:30	OG		Washing flask that was brought out from the room		
9:35	C		Sleeping on mother's lap		
9:40	M	C	Lying down child on the bed in the room		
9:40	M		Checking the fire wood		
9:45	OG		Bringing water into the house from the well outside in a metal bucket		
9:45	M		Rewashing the flask the daughter washed earlier		
9:50	OG		Taking her bath		
9:50	M		Checking the firewood		
9:55	M	HB	Chatting with husbands brother		
10:00	M		Mixing groundnut paste with water to make a watery mixture.	Groundnut	
10:05	M		Adding guinea corn flour into the groundnut mixture and mixing well to become smooth.	Guinea corn and ground nut	
10:10	M	HB	Chatting with husband's brother		
10:15	M		Adding guinea corn and groundnut mixture into the boiling water on the fire and stirring to make kunu		
10:15	C		Sleeping on the bed in the room		
10:20	M		Still stirring the kunu to cook well		
10:25	M		Mixing sugar and tamarind in a bowl		
10:30	M		Adding the sugar and tamarind and mix into the kunu on the fire	Kunun gyada	
10:35	M		Kunu is ready to be served	Kunun gyada	
10:35	M		Carrying the pot of kunu to the veranda	Kunun gyada	
10:35	M	OG	Chatting with the older daughter who is dressing up for school		
10:40	M		Serving the kunu in a large bowl	Kunun gyada	
10:40	M		Stirring the kunu to cool	Kunun gyada	

TIME	ACTOR INITIALS	RECIPIENT INITIALS	WHAT HAPPENED	FOOD	Reactivity
10:45	M		Still stirring the kunu to cool	Kunun gyada	
10:50	M	OG	Chatting with the older daughter while stirring the kunu	Kunun gyada	
10:50	C		Still sleeping		
10:55	OG		Leaving for school carrying a bag		
10:55	M		Still stirring the kunu to cool	Kunun gyada	
10:55	C		Still sleeping in the room		
11:00	C		Still sleeping in the room		
11:05	M		Serving kunu into the flask	Kunun gyada	
11:10	M		Sweeping the veranda		
11:15	M		Washing her hands		
11:20	M		Sitting under the shade on the mat		
11:25	M	G	Chatting with sister under the shade		
11:30	M	G	Chatting with sister		
11:35	C		Wakes up crying		
11:35	G	C	Carrying the child from the room		
11:35	G	C	Giving the child water to drink in a cup from the clay pot in front of the room	Water	
11:40	M	C	Carrying child on her laps. Playing with him		
11:45	M	C	Giving the child breast but he refused to breastfeed	Breast milk	
11:50	M	C	Carrying the child on her laps		
11:50	M	G	Chatting with sister under the shade		
11:55	G	C	Carrying the child on her lap showing him something on mobile phone		
11:55	M		Serving kunu from flask into a small cup	Kunun gyada	
12:00	M		Goes into room and brings MNP. Adding the MNP into the kunu ensuring the child did not see the	Kunun gyada	

TIME	ACTOR INITIALS	RECIPIENT INITIALS	WHAT HAPPENED	FOOD	Reactivity
			MNP		
12:00	M		Stirring the kunu to ensure the MNP dissolves well into the kunu	Kunun gyada	
12:05	M	C	Feeding the child the kunu mixed with MNP on her laps. Feeding him with the cup	Kunun gyada	
12:06	C		Drinking the kunu from the cup with ease	Kunun gyada	
12:07	C		Crying and refusing to take more kunu from the mother	Kunun gyada	
12:08	M	C	Forcefully giving the child the rest of the kunu. He gulps down whenever it enters his mouth but is crying	Kunun gyada	
12:09	M	C	Still feeding the child the kunu	Kunun gyada	
12:10	M	C	Washing childs hands and mouth after he finishes the whole kunu that was served into the cup	Kunun gyada	
12:15	M	C, G	Carrying child on her laps while chatting with sister		
12:20	G		Lying down on the ground under the shade chatting with the mother		
12:25	C	OB	Running towards brother when he came in and hugging him		
12:30	OB		Drinking water from the clay pot in front of the room		
12:30	M		Serving kunu into a bowl for the older son	Kunun gyada	
12:35	M		Lying down on the veranda chatting with sister		
12:35	G		Lying down in the room chatting with sister		
12:35	B		Playing near the older son who is drinking his kunu		
12:35	OB		Drinking kunu from the bowl with a large spoon		
12:40	B		Playing around the veranda. Enters room comes out and enter the other room too. Playing with		

TIME	ACTOR INITIALS	RECIPIENT INITIALS	WHAT HAPPENED	FOOD	Reactivity
			the curtain as he comes out from the room.		
12:45	M	G	Chatting with sister		
12:50	B		Sitting beside mother under a tree in the veranda		
12:55	B	OB	Playing with brother while he's drinking kunu	Kunun gyada	
12:55	OB		Licking the bowl after drinking the whole kunu	Kunun gyada	
13:00	OB		Washing his hands and mouth with water after dropping the bowl in the washing area		
13:05	OB	B	Playing with child in the veranda beside the mother		
13:10	M	G	Chatting with sister		
13:15	M	CL	Chatting with community leader who came in		
13:20	M	CL	Chatting with community leader		
13:25	M		Goes into room and bring out a bottle of oil		
13:25	M	CL	Handing the bottle to the community leader and collecting money		
13:30	CL		Bringing back the bottle chatting with the mother		
13:30	OB	B	Playing on the veranda		
13:35	B		Lying down on the veranda		
13:35	OB	B	Tickling the child while lying down on his back		
13:40	M		Putting more oil in the bottle the community leader brought back		
13:45	M	OB	Scolding the older son for playing roughly with the child		
13:50	OB	B	Playing with child. Making car sound while the child is laughing		
13:55	B		Drawing lines on the wall by the		

TIME	ACTOR INITIALS	RECIPIENT INITIALS	WHAT HAPPENED	FOOD	Reactivity
			room's entrance with a chalk		
14:00	B		Playing with the older son by the room door		
14:05	OB	B	Playing with child on the veranda		
14:05	M		Lying down on the veranda		
14:10	H		Listening to the radio outside the room and under a shade		
14:15	H	M	Chatting with the mother		
14:20	OB	B	Running around the house with the child		
14:25	H	B	Carrying child on his lap while listening to the radio		
14:30	H		Getting water from the well to take his bath. Back from farm earlier but rested first		
14:35	H		Taking his bath		
14:35	B		Sitting beside the mother playing		
14:40	B		Playing beside the mother with chalk		
14:45	OB		Lying down beside the mother on the veranda		
14:50	H	M	Chatting with the mother		
14:55	M	B	Playing with the child		
15:00	H	M	Chatting with the mother		
15:05	M	B	Breastfeeding the child while lying down	Breast milk	
15:10	M	H, B	Chatting with the husband while breastfeeding the child	Breast milk	
15:15	M	B	Breast feeding the child	Breast milk	
15:20	M	B	Breast feeding the child	Breast milk	
15:25	H	M	Chatting with the mother		

TIME	ACTOR INITIALS	RECIPIENT INITIALS	WHAT HAPPENED	FOOD	Reactivity
15:30	B		Sleeping on mother's lap		
15:35	M	B	Child sleeping on the veranda and covered with wrapper		
15:40	M		Starting fire		
15:45	M		Starting fire		
15:50	M		Starting fire		
15:55	M		Waiting for water to boil before adding guinea corn flour and make a meal		
16:00	M		Adding guinea corn flour into boiling water on fire to make meal	Guinea corn, water	
16:05	M		Stirring to ensure meal is smooth	Guinea corn, water	
16:10	M		Adding more guinea corn flour into meal	Guinea corn, water	
16:15	M		Covering meal to let it cook well	Tuwon dawa/ guinea corn meal	
16:20	M		Meal is ready to be served	Guinea corn meal	
16:20	M		Serving meal into large flask	Guinea corn meal	
16:25	M		Washing hands after preparing meal		
16:30	M		Adding water into another pot on fire	Water	
16:35	M		Washing pumpkin which is already peeled and put into the pot on fire	Pumpkin	
16:35	M		Cutting pumpkin into small pieces	Pumpkin	
16:40	M		Adding pumpkin into pot on fire	Pumpkin	

TIME	ACTOR INITIALS	RECIPIENT INITIALS	WHAT HAPPENED	FOOD	Reactivity
16:40	M	B	Checking on sleeping child		
16:45	M		Cutting pumpkin leaves in a bowl	Pumpkin leaves	
16:50	M		Cutting okro in a separate bowl	Okro	
16:55	M		Adding okro into pot on fire	Okro, pumpkin	
16:55	M		Washing pumpkin leaves and kept aside	Pumpkin leaves	
17:00	M		Checking firewood		
17:05	M		Adding pumpkin leaves into pot on fire	Pumpkin leaves	
17:05	B		Still sleeping on the veranda		
17:10	M		Adding groundnut paste mixed with water into pot on fire	Groundnut paste	
17:10	M		Stirring the soup to ensure all ingredients mix well	Pumpkin soup	
17:15	M		Adding pepper, salt and other ingredients into soup	Pumpkin soup	
17:15	M		Tasting to ensure salt is adequate	Pumpkin soup	
17:20	M		Soup is ready to be served		
17:20	M		Keeping the pot of soup aside	Pumpkin soup	
17:25	M		Sweeping the cooking area		
17:30	M		Washing the utensils used in cooking		
17:35	M	B	Checking the child		
17:35	B		Awake and just lying down on the wrapper under the verander		
17:35	M	B	Carrying the child		
17:40	M	B	Bathing the child		

TIME	ACTOR INITIALS	RECIPIENT INITIALS	WHAT HAPPENED	FOOD	Reactivity
17:45	M	B	Bathing the child		
17:50	M	B	Dressing the child		
17:55	M	B	Sitting on the mat on the veranda carrying child on her laps		
17:55	M	B	Breastfeeding the child	Breast milk	
18:00	M	B	Breastfeeding the child	Breast milk	
18:05	M	B	Breastfeeding the child	Breast milk	

TIME	ACTOR INITIALS	RECIPIENT INITIALS	WHAT HAPPENED	FOOD	Reactivity

TIME	ACTOR INITIALS	RECIPIENT INITIALS	WHAT HAPPENED	FOOD	Reactivity

TIME	ACTOR INITIALS	RECIPIENT INITIALS	WHAT HAPPENED	FOOD	Reactivity

TIME	ACTOR INITIALS	RECIPIENT INITIALS	WHAT HAPPENED	FOOD	Reactivity

TIME	ACTOR INITIALS	RECIPIENT INITIALS	WHAT HAPPENED	FOOD	Reactivity

PARTICIPANT ID: _________

Primary Questions (Complete after you complete data collection):

From what you have seen observing THIS HOUSEHOLD, please answer the following questions in detail:

Question	Write your detailed response here
1. In this household, what would be the best way(s) to give nutritional supplements such as an MNP to young children to ensure compliance?<br><br>Why?	The best way to ensure compliance is for the mother to ensure that time is fixed for giving the MNP
2. In this household, who is favored during meal preparation or feeding?<br><br>How is this favoritism expressed?	The parents are favoured during meal preparation because the food prepared was in a solid state even though a semi-solid food was prepared.<br>It was prepared just for administering the MNP and the older children drank it too.
3. How could we promote the MNP supplement for this family that would attract their attention?	This can be through the mother and the older daughter.<br>The father can also be an influence because he’s the one who has final say in decision in the household.<br>The father was listening to the radio, this can also be used in

Who would they listen to?	promoting the product. The community leader too can influence them because he is respected and the woman spoke highly respectful to him.
4. In this household, what would be the best ways to promote the MNP but limit how much it would be shared?<br><br>Why?	The best way to limit the sharing is through constant reminder to the mother on the implication of sharing the MNP because she will be reminded about the benefits of the MNP and if it is shared, it can neither benefit the child that its meant for and the person shared with.
5. What other issues and concerns did you see with this family that are important for us to consider with regard to introduction of an MNP?<br><br>Why?	There is good hygiene in the household. They all washed hands before and after meal.<br>The mother washed her hands before preparing the meals and after. She also swept the household and the cooking area after meal. Utensils were washed and kept in a clean place.
6. Based on your experience observing this child today, do you believe reactivity negatively impacted your ability to see the food-related behaviors of the child?<br><br>Please explain your answer in detail.	Reactivity did not affect behavior of the child.<br>The mother admitted to having difficulties feeding the child in recent days but it is due to fever which is being treated for.
